# The Use of Physiotherapy among Patients with Subacromial Impingement Syndrome: Impact of Sex, Socio-Demographic and Clinical Factors

**DOI:** 10.1371/journal.pone.0151077

**Published:** 2016-03-08

**Authors:** David Høyrup Christiansen, Poul Frost, Lars Henrik Frich, Deborah Falla, Susanne Wulff Svendsen

**Affiliations:** 1 Danish Ramazzini Centre, Department of Occupational Medicine, Regional Hospital West Jutland—University Research Clinic, Herning, Denmark; 2 Danish Ramazzini Centre, Department of Occupational Medicine, Aarhus Hospital, Aarhus University Hospital, Aarhus, Denmark; 3 Orthopedic Department, Odense University Hospital, Odense, Denmark; 4 Center for Anesthesiology, Emergency and Intensive Care Medicine, University Hospital Göttingen, Göttingen, Germany; 5 Institute for Neurorehabilitation Systems, Bernstein Focus Neurotechnology (BFNT) Göttingen Bernstein Center for Computational Neuroscience (BCCN), University Medical Center Göttingen Georg-August University, Göttingen, Germany; Fraunhofer Research Institution of Marine Biotechnology, GERMANY

## Abstract

**Background:**

Physiotherapy with exercises is generally recommended in the treatment of patients with subacromial impingement syndrome (SIS).

**Objective:**

We aimed to investigate the use of physiotherapy in patients with SIS in Danish hospital settings as part of initial non-surgical treatment and after SIS-related surgery and to evaluate to which extent sex, socio-demographic and clinical factors predict the use of physiotherapy.

**Methods:**

Using national health registers, we identified 57,311 patients who had a first hospital contact with a diagnosis of ICD-10, groups M75.1–75.9, 1 July 2007 to 30 June 2011. Records of physiotherapy were extracted within 52 weeks after first contact (or until surgery), and for surgically treated patients within 26 weeks after surgery. Predictors of the use of physiotherapy after first contact and after surgery were analysed as time-to-event.

**Results:**

Within 52 weeks after first contact, 43% of the patients had physiotherapy and 30% underwent surgery. Within 26 weeks after surgery, 80% had a record of physiotherapy. After first contact and after surgery, exercise was part of physiotherapy in 65% and 84% of the patients, respectively. A public hospital contact, physiotherapy before hospital contact, administrative region, female sex, a diagnosis of other or unspecified disorders (M75.8-M75.9), and surgical procedure predicted higher use of physiotherapy. Low education level predicted slightly lower use of physiotherapy after first contact, but not after surgery.

**Conclusion:**

In patients with SIS in Danish hospital settings, physiotherapy was more often used after surgery than as part of initial non-surgical treatment. The use of physiotherapy was less common among men than women, whereas unequal use of physiotherapy in relation to education level was not noticeable. The use of physiotherapy with exercises in initial non-surgical treatment was relatively limited.

## Introduction

Initial treatment of subacromial impingement syndrome (SIS) and related shoulder disorders is predominantly non-surgical, comprising rest, non-steroidal anti inflammatory drugs, corticosteroid injections, and different modalities of physiotherapy, in particular exercises. In the case of symptoms persisting for more than three months, referral for orthopaedic evaluation is common [1]. Although high success rates have been reported for surgical interventions in SIS patients [2–4], studies have reported a 10–23% risk of permanent work disability within 2–5 years after surgery [5,6], with a higher risk in relation to low education level [5] and receiving health-related transfer payments before surgery [6].

Randomised controlled trials have indicated an effect of physiotherapy with exercises with respect to reducing pain and improving function in patients with SIS [7] and reducing the patients’ self-assessed need for surgery [8–10]. Thus, clinical guidelines recommend at least three months’ physiotherapy including exercises before surgery is considered [1,11]. Postoperative exercise therapy is also recommended [11], although the effectiveness is less documented [12–14]. The use of physiotherapy in clinical practice has been sparsely examined. After subacromial decompression surgery, a recent Finnish study found that 50% of 104 patients received physiotherapy before surgery, whereas 77% received postoperative exercise instructions [15]. It is unknown, whether the use of physiotherapy in patients with SIS is influenced by sex, socio-demographic and clinical factors; such information could be used to identify target areas to promote implementation of clinical guidelines.

The aim of this study was to investigate the use of physiotherapy in patients with SIS in Danish hospital settings as part of initial non-surgical treatment and after SIS-related surgery. A further aim was to analyse to which extent sex, socio-demographic and clinical factors predict the use of physiotherapy. Specifically, we hypothesised that low education level (a marker of socio-economic status) would predict lower use of physiotherapy which might in part explain why low education level has been found to be associated with a higher risk of permanent work disability after SIS-related surgery [5].

## Materials and Methods

### Design and registers

We conducted a nationwide register-based cohort study. Three health registers were used: 1) the Danish National Patient Register (NPR), which contains information on diagnosis, hospital, contact dates, surgery dates, surgical procedures, and physiotherapy for in- and outpatient contacts including nature of intervention received (advice/instruction, exercise therapy, manual therapy, electro- and thermotherapy) in public and private somatic hospitals in Denmark from the beginning of 1995 and 2003, respectively [16], 2) the Danish National Rehabilitation Register (NRR), which includes contact date and intervention received in municipal settings from the beginning of 2007 (http://sundhedsdatastyrelsen.dk/da/registre-og-services/om-de-nationale-sundhedsregistre/sygdomme-laegemidler-og-behandlinger/genoptraening), and 3) the Danish National Health Service Register (NHSR), which contains week-by-week information on physiotherapy interventions received in private primary care since 1990 [17] with the exception of self-paid therapy without reimbursement. Together, the three separate health registers cover all settings where physiotherapy is provided. Moreover, we used information on education level and health-related transfer payments from the Danish National Register on Public Transfer Payments (DREAM). DREAM includes week-by-week registration of all types of public transfer payments, emigration, death, and unemployment insurance fund membership for all Danish citizens since 1994 [18].

### Study population

The study comprised all patients aged 18–65, who received an International Classification of Diseases, 10^th^ revision, discharge diagnosis in groups M75.1-M75.9 (M75.1 rotator cuff syndrome, M75.2 bicipital tendinitis, M75.3 calcific tendinitis, M75.4 impingement syndrome, M75.5 bursitis, M75.8 other shoulder lesions, and M75.9 unspecified shoulder lesions) without a subordinate diagnosis of M75.0 (adhesive capsulitis), in a public or private hospital between 1 July 2007 and 30 June 2011. To examine the use of physiotherapy after surgery, we identified the sub-population, who had SIS-related surgery (one or more Danish Nordic Medico-Statistical Committee [NOMESCO] shoulder and upper-arm surgery codes in groups KNBA, KNBE-H, and KNBK-M) within 52 weeks after their first hospital contact. Patients were excluded if they were not living in Denmark, or had emigrated or had shoulder surgery before their first contact. In the study of the use of physiotherapy after surgery, patients were excluded if they had non SIS-related surgery codes. The study was authorised by the National Board of Health and the Danish Data Protection Agency (J. no. 2010-41-4316). In Denmark, register-based studies do not require approval by committees on biomedical research ethics, or informed consent.

### The use of physiotherapy

We extracted records of physiotherapy within 26 weeks before first hospital contact, within 52 weeks after first hospital contact (or until surgery), and–for surgically treated patients—within 26 weeks after surgery. The primary outcome was the use of physiotherapy defined as ≥1 record of physiotherapy 1) after first contact, or 2) after surgery. Moreover, based on randomised controlled trials, which have demonstrated effectiveness of supervised exercise therapy in patients with SIS [8,12,19], we used ≥5 records in the same setting to define more extensive physiotherapy (thought to reflect supervised exercise therapy). Interventions were categorised as exercise therapy, manual therapy, information/advice on self-training, and other physical modalities (e.g. electro- and thermotherapy). Information on treatment before first hospital contact was extracted to provide a comprehensive description of the use of physiotherapy.

### Explanatory variables

The primary explanatory variable was education level as indicated by unemployment insurance fund membership in the year of the first hospital contact. Education level was categorised into higher or medium-level education, vocational education and training, low education level, and no information on education level (including receipt of cash benefit) [5].

Other explanatory variables included sex, age at the date of first hospital contact classified as 18–35, 36–45, 46–55, and 56–65 years, physiotherapy within 26 weeks before first hospital, diagnostic group in terms of specified disorders (M75.1-M75.5) and other or unspecified disorders (M75.8-M75.9), the hospital’s position in one of five administrative regions, and whether the patient had a private or public hospital contact. Surgical procedures were classified as 1) exploratory procedures (KNBA), 2) procedures on synovia and ligaments (codes KNBE and KNBF), 3) procedures on bursae and tendons (KNBL and KNBM), and 4) acromioplasty (KNBG, KNBH, and KNBK); we also distinguished between open and arthroscopic surgery [5]. Furthermore, we included health-related transfer payments within the year before first hospital contact, which was categorised as none or minimal (<12 weeks of sickness benefit, vocational rehabilitation benefit, or cash benefit except if cash benefit was received due to unemployment), temporary (≥12 weeks of sickness benefit, vocational rehabilitation benefit, or cash benefit except if cash benefit was received due to unemployment), and permanent (disability pension, voluntary early retirement because this may be chosen for health reasons, or flex-job, which is an economically subsidised job offered in case of limited work capacity). These definitions have been used to quantify social and economic consequences of health-related disability in prospective cohort studies [20,21].

### Statistical analysis

We used generalised linear regression analysis with application of pseudo-values to estimate the risk ratio (RR) of physiotherapy within 52 weeks after first contact and within 26 weeks after surgery in two separate models [22]. The pseudo-value regression approach enabled us to treat shoulder surgery as a competing risk, thereby solving the problem that patients who received surgery within 52 weeks after first contact could not contribute with 52 weeks of observation with respect to initial non-surgical treatment, as having surgery could not be considered to be independent of the use of physiotherapy. Patients who died or emigrated during follow up were censored. To explore whether a more restrictive definition of SIS would change our results, the analyses were repeated including M75.1 (rotator cuff syndrome) and M75.4 (impingement syndrome) only. Furthermore, as the completeness of registration in the newly established NRR could differ across administrative regions, we performed post-hoc analysis excluding information on physiotherapy in municipal settings. Data was analysed using STATA version 13 (Stata Corporation, College Station, Texas).

## Results

### Characteristics of the study population

**Fig 1:**
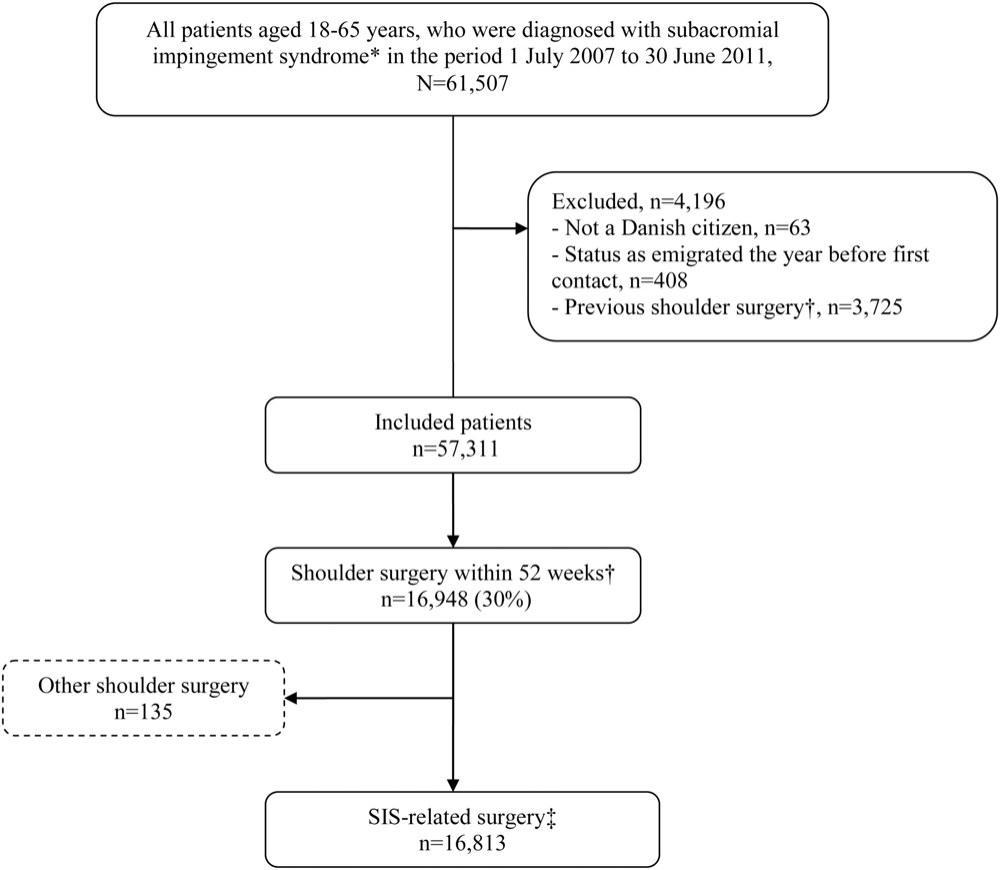
Flowchart of inclusion of patients in the study. * International Classification of Diseases, 10^th^ revision, codes M75.1-M75.9. † All KNB shoulder and upper-arm surgery codes from the Danish Nordic Medico-Statistical Committee (NOMESCO), ‡ Surgery codes KNBA, KNBE-H, and KNBK-M.

A total of 57,311 patients were included in the study (Fig 1).

Of these, 30% underwent shoulder surgery within 52 weeks after first hospital contact. The population for the study of postoperative use of physiotherapy comprised 16,813 patients after excluding patients who received non SIS-related shoulder surgery i.e., primary and secondary prosthetic replacement of joint of shoulder (codes KNBB and KNBC) n = 63; fracture surgery of shoulder and upper arm (code KNBJ) n = 22; transplantation in shoulder and upper arm (code KNBN) n = 3; operations for tumours of shoulder and upper arm (code KNBR) n = 10; operations for infection of tendons, joints and bone of shoulder and upper arm (code KNBS) n = 1, miscellaneous operations of shoulder and upper arm (code KNBT) n = 13 and removal of implants and external fixation devices from shoulder and upper arm (code KNBU) n = 23.

Table 1 presents characteristics of the included patients.

**Table 1:** Characteristics of patients with subacromial impingement syndrome (SIS) at first hospital contact 2007–2011 (n = 57,311), in total and according to education level.

Characteristics		Total		Higher or medium-level education		Vocational education and training		Low education level		No information on education level	
Sex											
	Female	28,266	(49)	7,302	(48)	9,315	(46)	9,564	(55)	2,085	(45)
	Male	29,045	(51)	7,838	(52)	10,852	(54)	7,845	(45)	2,510	(55)
Age group, years											
	18–35	9,575	(17)	1,965	(13)	3,671	(14)	2,424	(14)	1,515	(33)
	36–45	13,645	(24)	3,371	(22)	5,086	(25)	4,401	(25)	787	(17)
	46–55	18,719	(33)	5,202	(34)	6,271	(31)	6,210	(36)	1,036	(23)
	56–65	15,372	(27)	4,602	(30)	5,139	(25)	4,374	(25)	1,257	(27)
Health-related transfer payments											
	None or minimal	38,654	(68)	11,831	(78)	14,245	(71)	10,218	(59)	2,360	(51)
	Temporary	8,799	(15)	1,404	(9)	2,760	(14)	3,681	(21)	954	(21)
	Permanent	9,858	(17)	1,905	(13)	3,162	(16)	3510	(20)	1,281	(28)
Diagnosis											
	Specified disorders (M75.1-M75.5)	40,019	(70)	10,668	(70)	13,998	(69)	12,390	(71)	2,963	(64)
	Other or unspecified disorders (M75.8-M75.9)	17,292	(30)	4,472	(30)	6,169	(31)	5,019	(29)	1,632	(36)
Hospital of first contact											
	Private	15,521	(27)	4,574	(30)	6,289	(31)	3,760	(22)	898	(20)
	Public	41,790	(73)	10,566	(70)	13,878	(69)	13,649	(78)	3,697	(80)
Physiotherapy before first contact											
	No	42,750	(75)	11,200	(74)	14,823	(74)	12,989	(75)	3,738	(81)
	Yes	14,561	(25)	3,940	(26)	5,344	(27)	4,420	(25)	857	(19)
Administrative region											
	Capital Region of Denmark	11,805	(21)	3,630	(24)	4,084	(20)	2,780	(16)	1,311	(29)
	Region Zealand	7,401	(13)	1,842	(12)	2,600	(13)	2,386	(14)	573	(12)
	Region of Southern Denmark	15,361	(27)	3,855	(25)	5,330	(26)	5,046	(29)	1,130	(25)
	Central Denmark Region	16,282	(28)	4,203	(28)	5,926	(29)	4,981	(29)	1,172	(26)
	North Denmark Region	6,462	(11)	1,610	(11)	2,227	(11)	2,216	(13)	409	(9)

Values are numbers (%). Percentages do not always add up to 100 due to rounding.

The mean age was 47 years (SD 11). M75.4 (impingement syndrome) and M75.1 (rotator cuff syndrome) made up the largest part of the diagnoses in the SIS group (62%). The patients were distributed with 26% who had higher or medium-level education, 35% who had vocational education and training, 30% who had low education level, and 8% who had no information on education level. The majority of patients with low education level were female (55%). The percentage of patients who underwent surgery varied from 24% in Region of Southern Denmark to 39% in North Denmark Region. More patients with diagnoses of specified disorders (M75.1-M75.5) patients with other or unspecified disorders (M75.8-M75.9) (34% versus 20%, respectively).The percentage who had surgery was 28% for patients with higher or medium-level education, 30% for patients with vocational education and training, 33% for patients with low education level, and 21% for patients with no information on education level.

### The use of physiotherapy

Within 52 weeks after first hospital contact, 43% had physiotherapy and 21% (i.e., half of those who received physiotherapy) had more extensive physiotherapy. When physiotherapy before first contact was taken into account, the total numbers increased to 54% and 31%, respectively. Among patients, who ended up having surgery during the follow up period, 45% had at least one record of physiotherapy before or after first hospital contact. Postoperative physiotherapy was received by 80% of the patients, with 52% receiving more extensive physiotherapy.

**Fig 2:**
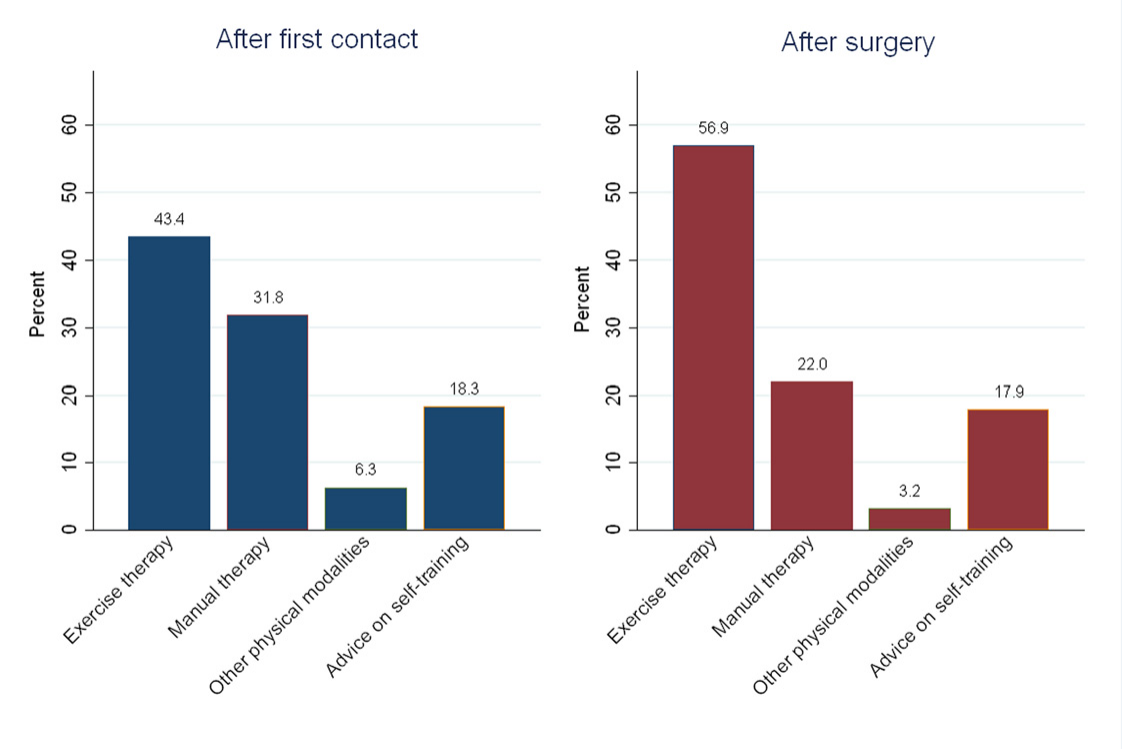
Overall distribution of codes of physiotherapy interventions after first contact and after surgery. **After first contact:** Based on a total of 339,050 codes of physiotherapy interventions in patients who received physiotherapy within 52 weeks after first hospital contact (n = 24,452). **After surgery:** Based on a total of 233,630 codes of physiotherapy interventions in patients who received physiotherapy within 26 weeks after SIS-related surgery (n = 13,394). Of note, more than one code of physiotherapy intervention can be recorded per contact.

Fig 2 displays the overall distribution of codes of physiotherapy interventions, of which exercise therapy made up the largest part.

After first contact and after surgery, respectively, 62% and 75% of all recorded intervention codes indicated either exercise therapy or advice on self-training. Focussing on the codes that were registered for each patient (rather than the overall distribution of codes), we found that at least one exercise therapy intervention code was recorded for 65% of the patients who received physiotherapy after first contact, and that at least one code of exercise therapy and/or advice on self-training was recorded for 95% of the same group of patients. After surgery, the corresponding values were 84% and 98%.

### Predictors of physiotherapy

The RR of the use of physiotherapy after first hospital contact is presented in Table 2.

**Table 2:** Risk ratios (RR) of the use of physiotherapy within 52 weeks after first hospital contact in relation to sex, socio-demographic and clinical factors in patients with subacromial impingement syndrome (n = 57,311).

			%				
				RR _crude_	RR _adjusted_*	95% CI	
Level of education		** **					
	Higher or medium-level		43	1.00	1.00		-
	Vocational education and training		43	1.01	1.01	0.99	1.04
	Low level		42	0.97	0.95	0.93	0.98
	No information		42	0.98	0.95	0.91	0.98
Sex							
	Male		38	1.00	1.00		-
	Female		47	1.22	1.16	1.13	1.18
Age group, years							
	18–35		47	1.00	1.00		-
	36–45		42	0.90	0.92	0.89	0.95
	46–55		43	0.92	0.93	0.91	0.95
	56–65		40	0.85	0.88	0.86	0.91
Health-related transfer payments							
	None or minimal		42	1.00	1.00		-
	Temporary		47	1.12	1.01	0.99	1.03
	Permanent		42	1.00	0.99	0.96	1.01
Diagnosis							
	Specified disorders (M75.1-M75.5)		41	1.00	1.00		-
	Other or unspecified disorders (M75.8-M75.9)		46	1.11	1.15	1.13	1.17
Hospital of first contact							
	Private		34	1.00	1.00		-
	Public		46	1.37	1.39	1.36	1.43
Physiotherapy before first contact							
	No		38	1.00	1.00		-
	Yes		57	1.52	1.49	1.46	1.51
Administrative region							
	Capital Region of Denmark		50	1.00	1.00		-
	Region Zealand		40	0.81	0.81	0.78	0.83
	Region of Southern Denmark		50	0.99	0.97	0.94	0.99
	Central Denmark Region		37	0.74	0.73	0.71	0.75
	North Denmark Region		31	0.61	0.64	0.61	0.66

% = Percentages of patients who had physiotherapy, CI = confidence interval.

*Adjusted for all other variables shown.

Weak negative associations were found for patients with low education level and with no information on education level as compared to patients with higher or medium-level education. Explanatory variables demonstrating positive associations included female sex, physiotherapy before first hospital contact, a public hospital contact, and to a lesser extent, a diagnosis of unspecified or other shoulder disorders. Older age was associated with lower use of physiotherapy. Using a more restrictive definition of SIS (M75.1 and M75.4) did not change the results. Large variation was found between the administrative regions, with particularly low use of physiotherapy in North Denmark Region (Table 2). Post-hoc analysis excluding information on physiotherapy in municipal settings (NRR) did not change the results in relation to regional variations (results not shown).

Among 16,813 patients who had SIS-related surgery, 97% of the operations were performed arthroscopically. The most frequent surgical procedure was acromioplasty (80%), followed by procedures on synovia and ligaments (12%), procedures on bursae and tendons (6%), and explorative procedures (2%). Table 3 displays the RR of the use of postoperative physiotherapy in relation to sex, socio-demographic and clinical variables.

**Table 3:** Risk ratios (RR) of the use of physiotherapy within 26 weeks after surgery in relation to sex, socio-demographic and clinical factors in patients who had SIS-related surgery (n = 16,813). *

			%	RR _crude_	RR _adjusted_†	95% CI	
Level of education							
	Higher or medium level		79	1.00	1.00		-
	Vocational education and training		79	1.00	1.00	0.98	1.02
	Low level		81	1.03	1.00	0.99	1.02
	No information		81	1.03	0.99	0.96	1.01
Sex							
	Male		77	1.00	1.00		-
	Female		82	1.07	1.06	1.05	1.07
Age group, years							
	18–35		78	1.00	1.00		-
	36–45		77	1.00	1.00	0.97	1.02
	46–55		80	1.03	1.02	0.99	1.04
	56–65		82	1.06	1.03	1.01	1.06
Health-related benefits							
	None or minimal		78	1.00	1.00		-
	Temporary		82	1.06	1.01	0.99	1.03
	Permanent		83	1.06	1.00	0.98	1.01
Diagnosis							
	Specified disorders (M75.1-M75.5)		80	1.00	1.00		-
	Other or unspecified disorders (M75.8-M75.9)		77	0.96	1.03	1.02	1.05
Hospital of surgery							
	Private		66	1.00	1.00		-
	Public		90	1.37	1.31	1.29	1.34
Surgical procedures							
	Exploratory procedures		68	1.00	1.00		-
	Procedures on synovia and ligaments		61	0.90	1.00	0.93	1.07
	Procedures on bursae and tendons		82	1.20	1.14	1.07	1.22
	Acromioplasty		83	1.22	1.10	1.04	1.17
Surgical category							
	Arthroscopic		80	1.00	1.00		-
	Open		84	1.06	1.07	1.03	1.10
Administrative region							
	Capital Region of Denmark		84	1.00	1.00		-
	Region Zealand		88	1.04	1.00	0.99	1.02
	Region of Southern Denmark		81	0.96	0.91	0.89	0.92
	Central Denmark Region		82	0.97	0.93	0.91	0.94
	North Denmark Region		60	0.71	0.76	0.73	0.78

% = Percentage who had physiotherapy, CI = Confidence interval

* A total of 135 patients without a relevant operation code were excluded

†Adjusted for all other variables shown

The use of postoperative physiotherapy was not related to education level. The results did not change when a more restrictive definition of SIS (M75.1 and M75.4) was applied. Surgery in a public hospital was the strongest positive predictor for postoperative physiotherapy, while administrative regions other than Capital Region of Denmark and Region Zealand were negative predictors–in particular, this was true of North Denmark Region (Table 3). Significant regional variations persisted when information on physiotherapy in municipal settings was excluded, but the difference with respect to North Denmark Region was reduced (results not shown).

## Discussion

This nationwide register study evaluated the use and predictors of physiotherapy in the management of patients with SIS. Within one year after first hospital contact, but before any surgical treatment, 43% of the patients had physiotherapy. When adding physiotherapy within half a year before first hospital contact, the percentage increased to 54%. After surgery, the majority of patients received physiotherapy. Most physiotherapy interventions included exercise therapy. A public hospital contact, physiotherapy before hospital contact, administrative region, a diagnosis of other or unspecified disorders (M75.8-M75.9), and surgical procedure predicted higher use of physiotherapy. Male sex predicted lower use of physiotherapy, while older age predicted lower use of physiotherapy after first contact and slightly higher use of physiotherapy after surgery. Patients with low education level were slightly less likely to receive physiotherapy after first contact, whereas the use of physiotherapy after surgery was not related to education level.

Since health registers in Denmark are population based and do not require consent from patients, selection bias due to non-participation and loss to follow-up is avoided [23]. We chose to include all diagnoses in group M75 (shoulder lesions) with the exception of M75.0 (adhesive capsulitis of shoulder). It could be argued that a more specific diagnostic definition would have been appropriate. However, the diagnostic label of SIS is based on clinical findings and symptoms caused by various underlying pathologies and mechanisms [24,25] and criteria to define SIS are not consistently applied in clinical practice or clinical trials [26,27]. In a Cochrane review of diagnostic labels and definitions of study populations, the authors concluded that most trials could only be broadly categorised as studying rotator cuff/impingement syndrome or adhesive capsulitis [26]. Additionally, physiotherapy with exercises may be relevant for all of the diagnoses, which we included, and sensitivity analyses restricted to M75.1 (rotator cuff syndrome) and M75.4 (impingement syndrome) did not change the results. Our choice was further supported by the finding that 20% of the patients with diagnoses of other or unspecified disorders (M75.8-M75.9) later received SIS-related surgery (versus 34% of the patients with specified disorders) and that only a very small percentage of the total cohort received non SIS-related surgery.

The completeness and validity of the registered physiotherapy interventions should also be considered. NPR data has not been evaluated in this respect. Registration in the NHSR at each contact in private primary care is necessary for the physiotherapist to get a full fee, and therefore the coverage of registration is assumed to be high [17]. Still, physiotherapists in private primary care, who provide self-paid therapy without reimbursement, do not report to the NHSR. According to the Danish Physiotherapy Association, this was true of an average of 12% of all registered physiotherapists in Denmark in the years 2008 to 2011; treatments by these physiotherapists could therefore not be accounted for in the present investigation.

The validity and completeness of the NRR have not been investigated, and although it is compulsory for the municipalities to report to the NRR, the completeness may differ between administrative regions. The information in the NRR and NHSR does not include clinical diagnoses, which means that we could not sort out physiotherapy received in relation to other conditions (e.g. neck or back pain). To minimise such validity problems, we limited the time windows for registration of physiotherapy to 26 weeks before and 52 weeks after first hospital contact for SIS.

We did not reduce the time window after first hospital contact further because this might lead to underestimation of the use of physiotherapy. It should be noted that although some of the patients in our study did not have any record of physiotherapy, we do not know whether these patients were managed adequately, e.g. with corticosteroid injections or information on self-training

Different completeness of registration of physiotherapy in the NRR across administrative regions may have led to an overestimation of regional variations. Post-hoc analysis excluding information on physiotherapy from municipal settings (NRR) did not change the result regarding regional variations in physiotherapy after first hospital contact, but regional variations in postoperative physiotherapy were reduced–possibly because physiotherapy in municipal settings is more often used after surgery (a previous hospital contact is a prerequisite for physiotherapy in municipal settings). Thus, the regional variations in postoperative physiotherapy should be interpreted with caution. Altogether, we most likely underestimated the use of physiotherapy, but to a limited extent.

The strongest predictor of the use of physiotherapy after first hospital contact was previous physiotherapy, whereas we would have expected physiotherapy to be used more often in patients who had not had physiotherapy previously–especially before the decision to proceed to surgery. We do not know which proportion of the patients were advised to have physiotherapy after first hospital contact, but patients’ preferences seem to be a likely part of the explanation why the same patients tended to have physiotherapy before and after first hospital contact. Patient preferences may also be reflected by the lower use of initial physiotherapy in older age and by the fact that men were less likely to have physiotherapy than women. Clinical variables that are not available for register-based research such as clinical examination findings, duration of symptoms, intensity of pain, nocturnal pain, and functional limitations may also play a part in the choice of treatment, as well as surgeons’ preferences. Unequal use of physiotherapy in relation to education level was not noticeable, although low education level did predict slightly lower use of physiotherapy after first hospital contact. Thus, the relatively poor prognosis that has been found for surgical shoulder patients with low education level [5] does not seem to be related to unequal access to postoperative physiotherapy.

Our results concerning the use of physiotherapy among surgically treated patients were in accordance with findings from previous studies that have been conducted in single orthopaedic departments [15,28]. The percentage of patients, who received physiotherapy after first hospital contact but before any surgery in our study (45%), was slightly lower than reported from Finland (53%) [15], which may be related to differences in study design and methods. In the Finnish study, information on treatment before surgery was collected by questionnaire one year after surgery and the timeframe for questions on physiotherapy treatment was not reported. The percentage who received physiotherapy after surgery in our study was 80%, and the treatment typically included exercises. Postoperative exercise instructions within one year were reported by 77% in the Finnish study. In a similar retrospective Swedish study of 95 patients, 97% reported to have received physiotherapist-supervised exercises after surgery [28].

Longitudinal population-based health registers allow treatment application to be monitored and provide a valuable data source for research. The relatively large regional variations and the finding that less than half of the patients with SIS received physiotherapy as part of initial non-surgical treatment suggest an untapped treatment potential, which may not be limited to countries with similar health care and social systems to those in Denmark. To optimise the quality of care, we suggest intensified efforts to inform patients that in general, surgery for SIS should be considered only after physiotherapy with exercises. The observed regional variation in the utilisation of physiotherapy may reflect differences in treatment policy and accessibility to self-paid physiotherapy. In 2013, national clinical guidelines for the diagnosis and treatment of SIS were issued by the Danish Health and Medicines Authority [11]. These guidelines included recommendations of at least 3 months’ non-surgical treatment with the inclusion of physiotherapist-guided exercise before proceeding to surgery. Findings from this study could serve as a comparator when evaluating the impact of the guidelines in years to come.

## Conclusion

Physiotherapy was more often used after surgery than as part of initial non-surgical treatment, where less than half of the patients received physiotherapy. The use of physiotherapy was less common among men than among women, whereas unequal use of physiotherapy in relation to education level was not noticeable. Overall, the use of physiotherapy with exercises in initial non-surgical treatment was relatively limited.
